# Supplementary material for: Gene dosage of PDR16 modulates azole susceptibility in Candida auris
Source: Microbiol Spectr. 2025 Mar 25;13(5):e02659-24. doi: 10.1128/spectrum.02659-24 (PMC12054019; doi:10.1128/spectrum.02659-24)
Supplement: Supplemental material — Fig. S1; Table S1. [file spectrum.02659-24-s0001.docx]

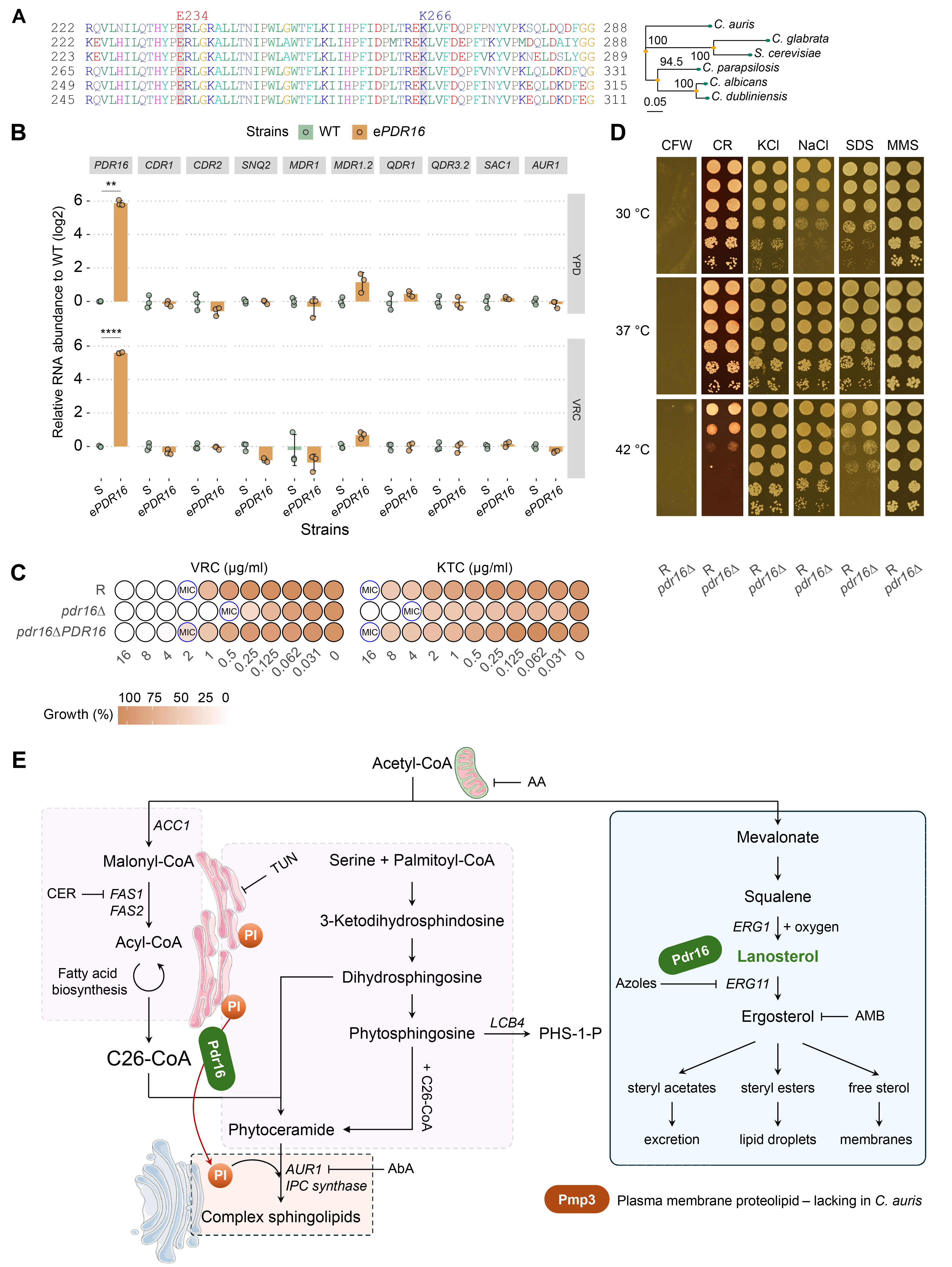


**Supplementary Fig. 1. A.** Phylogenetic tree was constructed with the neighbor-joining tree estimation with bootstrap 1000. The scale bar indicated evolutionary distance. Multiple sequence alignment of Pdr16 homologues using the clustal 2.1 tool. E234 and K266 are critical residues for PI-binding. **B.** mRNA abundance of selects drug resistance genes in ePdr16 and WT strains. Data normalized with the housekeeping gene *ACT1*. **C.** Dose response MIC assays demonstrated that the complemented reintegration strain restored resistance to voriconazole and ketoconazole. **D.** Agar plate spotting assays were conducted at different temperatures and with stress agents for strains R and *pdr16∆*. The inhibitors were used at the same concentrations as described in Fig. 1C. **E.** Proposed cellular pathways and biological processes affected by increased *PDR16* gene dosage. Phosphatidylinositol (PI); Phytosphingosin-1-Phosphate (PHS-1-P); Inositol Phosphoryl Ceramides (IPC). *p < 0.01, ****p <0.0001 were determined using a t-test followed by the Benjamini-Hochberg method for multiple comparisons. Figure 1C is a modified drawing retrieved from Servier Medical Art (https://smart.servier.com/).

**Table S1. Primers used in this study**

| ID | Name | Sequence | Purpose |
| --- | --- | --- | --- |
| 362 | NEUT1_55 | GTTGTGAACAGAACAAAGAG | to amplify NEUT1-5flank and *ENO1* promoter from plasmid pCauR_NEU_dTOM_NAT |
| 363 | ENO1_rev | GATGAAAATTAAGTTTGGATAGGG | to amplify NEUT1-5flank and *ENO1* promoter from plasmid pCauR_NEU_dTOM_NAT |
| 366 | NAT1-fw | TGCTTGCCTCGTCCCCGC | to amplify *NAT1* marker and NEUT1-3flank |
| 367 | NAT1-rev | ggatgcgacgtgcaagattacCC | to amplify *NAT1* marker and NEUT1-3flank |
| 385 | orf-pdr16-fw | CCCTATCCAAACTTAATTTTCATC*ATGCTTTTCAAAAAGGACAAA* | to amplify *PDR16* ORF from gDNA of C. auris |
| 386 | orf-pdr16-rev | GCGGGGACGAGGCAAGCA*TATGTGAGAAGACTGGAGTC* | to amplify *PDR16* ORF from gDNA of C. auris |
| 368 | NEUT_33 | CCAATGAAGCCATTGAGTTG | for overlapping PCR (in a pair with NEUT1_55) |
| 369 | upPDR16_check5fw | ACTCGGCACACTACTCTCC | to confirm 5 flank integration |
| 387 | upPDR16_check5rev | TTCTTCAGGCACCTTTGACTC | to confirm 5 flank integration |
| 371 | upPDR16_check3fw | GGTACTGCTTCCGATGGTG | to confirm 3 flank integration |
| 372 | upPDR16_check3rev | GCAGCGGTTACGAGTATGC | to confirm 3 flank integration |
| 394 | SNQ2qPCRfw | ATGTTCCGGTGCAAACCTTG | for qPCR - B9J08_001125 |
| 395 | SNQ2qPCRrev | CATCACCACTGACGATGCTC | for qPCR - B9J08_001125 |
| 396 | CDR1qPCRfw | GGCTCTTCTGTGGTCTCTGT | for qPCR - B9J08_000164 |
| 397 | CDR1qPCRrev | TAGCAGGAGTCATAGCAGCC | for qPCR - B9J08_000164 |
| 398 | CDR2qPCRfw | TGACACCGGCATCACTTTTG | for qPCR - B9J08_002451 |
| 399 | CDR2qPCRrev | TGATCCAACGACACCATCCA | for qPCR - B9J08_002451 |
| 400 | MDR1qPCRfw | CAGCAACCAGGAAGTCGTTC | for qPCR - B9J08_003981 |
| 401 | MDR1qPCRrev | CCGTCCAGCCAAAGATGAAG | for qPCR - B9J08_003981 |
| 402 | PDR16qPCRfw | CGATGCTGACAATGCCGTAA | for qPCR - B9J08_004982 |
| 403 | PDR16qPCRrev | ACATGGTCTGGAGTCGTTGT | for qPCR - B9J08_004982 |
| 406 | MDR1.2_004113_fw | AGACGACAATATCTGGGGCC | for qPCR - B9J08_004113 |
| 407 | MDR1.2_004113_rev | TGGGCTCTCTGAGGAACATC | for qPCR - B9J08_004113 |
| 408 | QDR1_005492_fw | GGGTTCGTCTTGATTTGGCA | for qPCR - B9J08_005492 |
| 409 | QDR1_005492_rev | TATTACCACGCCAACAACGC | for qPCR - B9J08_005492 |
| 410 | QDR3.2_005403_fw | CTGGCATCAACGTTCTCTCG | for qPCR - B9J08_005403 |
| 411 | QDR3.2_005403_rev | CCGAGTTCTTTCTTGAGGCG | for qPCR - B9J08_005403 |
| 412 | SAC1_004042_fw | GGTCAGGCTTGGGAGATCAT | for qPCR - B9J08_004042 |
| 413 | SAC1_004042_rev | GAGTAAGCACACGAGACTGC | for qPCR - B9J08_004042 |
| 414 | AUR1_005313_fw | GTTCCTCTCGTGGCTTTTCC | for qPCR - B9J08_005313 |
| 415 | AUR1_005313_rev | AGTAGTGGTGCGTGAGGTAC | for qPCR - B9J08_005313 |
